# Dual-locus DNA metabarcoding reveals southern hairy-nosed wombats (*Lasiorhinus latifrons* Owen) have a summer diet dominated by toxic invasive plants

**DOI:** 10.1371/journal.pone.0229390

**Published:** 2020-03-06

**Authors:** Amanda Camp, Adam E. Croxford, Caroline S. Ford, Ute Baumann, Peter R. Clements, Stefan Hiendleder, Lucy Woolford, Gabrielle Netzel, Wayne S. J. Boardman, Mary T. Fletcher, Mike J. Wilkinson

**Affiliations:** 1 School of Animal and Veterinary Science and Davies Research Centre, The University of Adelaide, Adelaide, SA, Australia; 2 School of Agriculture, Food and Wine, The University of Adelaide, Adelaide, SA, Australia; 3 Wales Veterinary Science Centre, Aberystwyth, United Kingdom; 4 Australian Centre for Plant Functional Genomics, The University of Adelaide, Adelaide, SA, Australia; 5 School of Animal and Veterinary Science and Davies Research Centre, The University of Adelaide, Adelaide, SA, Australia; 6 Centre for Animal Science, The University of Queensland, Brisbane, Queensland, Australia; 7 Institute of Biological Environmental and Rural Sciences, Aberystwyth University, Aberystwyth, Wales, United Kingdom; Institute for Biological Research, SERBIA

## Abstract

Habitat degradation and summer droughts severely restrict feeding options for the endangered southern hairy-nosed wombat (SHNW; *Lasiorhinus latifrons*). We reconstructed SHNW summer diets by DNA metabarcoding from feces. We initially validated *rbcL* and *ndhJ* diet reconstructions using autopsied and captive animals. Subsequent diet reconstructions of wild wombats broadly reflected vegetative ground cover, implying local rather than long-range foraging. Diets were all dominated by alien invasives. Chemical analysis of alien food revealed *Carrichtera annua* contains high levels of glucosinolates. Clinical examination (7 animals) and autopsy (12 animals) revealed that the most degraded site also contained most individuals showing signs of glucosinolate poisoning. We infer that dietary poisoning through the ingestion of alien invasives may have contributed to the recent population crashes in the region. In floristically diverse sites, individuals appear to be able to manage glucosinolate intake by avoidance or episodic feeding but this strategy is less tractable in the most degraded sites. We conclude that recovery of the most affected populations may require effective *Carrichtera* management and interim supplementary feeding. More generally, we argue that protection against population decline by poisoning in territorial herbivores requires knowledge of their diet and of those food plants containing toxic principles.

## Introduction

Australia hosts the world’s most distinctive mammalian fauna but has suffered more extinctions in the past 200 years than any other nation [1]. Wombats are marsupials (family Vombatidae) that comprise three species: bare-nosed (*Vombatus ursinus*), northern hairy-nosed (*Lasiorhinus kreffii*) and southern hairy-nosed (*Lasiorhinus latifrons*). Wombats are the world’s largest burrowing herbivores and typically forage nocturnally. All species have declined significantly since the spread of European farming [2]. Fragmented southern hairy-nosed wombat (SHNW) populations occur in South Australia and Western Australia [3] and are considered ‘near-threatened’ on the IUCN red list [4]. Extensive population crashes in South Australia have been informally linked to overgrazing, drought and influx of invasive plants [5]. SHNWs also suffer from grazing competition [6] and sarcoptic mange [7].

SHNWs have adapted to extract sufficient water from their preferred grass diet to survive summer droughts [8] and so may be vulnerable to dietary change. Over recent decades habitats supporting SHNWs have suffered invasion by several alien plant species, most notably *Carrichtera annua*, *Asphodelus fistulosus*, *Moraea setifolia* and various species of *Medicago* (e.g. [9]). This has occurred at the expense of native grasses that previously flourished throughout summer [10]. This poses a dilemma for SHNW survival. Avoidance of alien species risks accelerating displacement of favored grasses. Conversely, if the SHNWs do graze on the invaders, their new food source may not hold sufficient water or nutrients to enable survival of summer. The new food may also be toxic.

SHNWs are thought to have a narrower diet than generalist herbivores [6], and although capable of broadening dietary choices under stress [11] may be ill-equipped to digest novel plant species or to tolerate any toxins they contain. It is therefore important to characterize their diet, especially during the dry summer months, when the species is most vulnerable.

Interest has been growing over recent years over the use of DNA metabarcoding approaches to reconstruct the diets of animals [12,13,14,15,16]. Diet reconstruction of predatory species typically make use of the universal animal barcode, cytochrome oxidase 1 (CO1) [15,16]. In the case of herbivore diets however, relatively few studies have used the core plant barcodes (*rbcL* and *matK*) for this purpose [17,18]. *MatK* is notoriously difficult to amplify in some plant groups [19] and its length (950p) is ill-suited to most metabarcoding methodologies [20]. Most researchers have therefore elected to work with *rbcL* (e.g. [18]) and/or shorter non-coding loci such as *trnL* [17,21]. Regardless of the marker choice made, it is important to recognize that no metabarcoding protocol is free from error. It is preferable to incorporate controls and (ideally) multiple loci when attempting to reconstruct diets using these approaches [22]. Here, we deploy *ndhJ* and *rcbL* metabarcoding on fresh SHNW scats to test the extent to which alien plants feature in their summer diet. We characterized the toxin content of alien plants eaten and surveyed SHNWs for diagnostic signs of poisoning.

## Materials and methods

### Statement on animal ethics

Veterinary and pathological investigations were conducted by qualified and registered veterinarians under University Animal Ethics Approval S-2011-196; S2011-197D and S2014-075, and DEW scientific permit Q25996-1. Permission for site access for Moorunde was granted from Dr Peter Clements, President of the Moorunde Wildlife Land Reserve Charitable Trust (which owns the land), and from Dr David Taggart for Kooloola and Portee (Owned by South Autralian Govt (SA); S.A. Dept of Environment and Water, Scientific Research Permit A26829-1).

### Sample collection

Plant and scat samples were collected for diet reconstruction during February-March 2013 from three South Australian locations: Moorunde (34.46382^o^S, 139.47087^o^E), Kooloola (34.53875^o^S, 139.58832^o^E) and Portee (34.46241^o^S, 139.47336^o^E). Scats were also collected from captive animals. Wild plant species were identified morphologically [23]. Relative species abundance was assessed at each site and reference voucher specimens collected for barcoding. Fresh scats were collected along North-South and East-West transects of 1.25km, and stored at -80°C.

### Plant phytochemistry

Plant leaf material was collected for phytochemical analysis from all three sites during February-March 2015. For *Carrichtera annua*, green emergent seedlings, adult plants and persistent dead materials with siliques containing seeds were sampled.

### Clinical and pathological investigations

Seven wombats in poor health (1 adult, 5 subadults, 1 juvenile) were presented for veterinary evaluation, all from Portee. Haematological and biochemical analyses were conducted at the local Veterinary Diagnostic Laboratory (VDL). One of these animals subsequently died and was autopsied. In addition, SHNWs from the same area, either found dead (e.g. road accident) or humanely euthanised on welfare grounds were subjected to autopsy and alimentary canal sampling. These animals originated from Kooloola (n = 2), Moorunde (n = 4), Portee (n = 7), and elsewhere from the Murraylands (n = 7). Ingested material from the stomach, intestine and/or internal scats of these animals was collected and stored at -80°C.

### DNA extraction

DNA of abundant plant species (S1 Table) was extracted from leaves using the Isolate II Plant DNA extraction kit (Bioline). DNA was also extracted from freeze-dried (~10 mg) autopsy samples and scats using the same kit, with protocol adjustments of 600 μl lysis buffer and 675 μl binding buffer, 30μl elution in PG buffer. Six scats were halved and independent DNA extractions made from both halves to assess intra-scat variability.

### Plant reference barcodes

Reference barcode PCRs were conducted in 25 μl mixtures comprising: 1x Biomix (Bioline); 200 pM of primers (ndhJ1+ndhJ4 [24]; rbcLa_f + rbcLa_rev, [25,26] and 25 ng of DNA. PCR conditions were: 94°C for 30 s, 40 cycles of 94°C 30 s, 54°C 60 s, 72°C 60 s and a final extension of 72°C 5 min. Products were purified using Nucleofast 96 PCR plates (Macherey-Nagel) and Sanger-sequenced. The forward and reverse reads were aligned, edited and trimmed in Geneious (V8.1, Biomatters). Further reference barcodes were recovered from the NCBI database for food plants of captive animals (S1 Text).

### NGS metabarcoding of scat/autopsy samples

A two-step PCR generated *rbcL* and *ndhJ* barcodes from scat/autopsy samples. First, the following Illumina sequences were added to the 5' end of primers:

F: TCGTCGGCAGCGTCAGATGTGTATAAGAGACAG,

R: GTCTCGTGGGCTCGGAGATGTGTATAAGAGACAG.

Initial amplification was performed in 20μl of: 1x MyFi Buffer, 1.6U MyFi Polymerase (Bioline), 200 pM of each forward and reverse primers, and 20 ng DNA. PCR conditions for *rbcL and ndhJ*: 95°C (1 min) followed by 35 cycles of 95°C 15 s, 55°C 15 s, 72°C 15 s. Products were purified using the Agencourt AMPure XP PCR Purification beads at a v/v ratio of 0.6x beads/PCR product.

Second PCR was performed in 12.5 μl volumes of: 1x MyFi Buffer (Bioline,); 0.4 nM of paired Nextera 96 Indices (Illumina); 1.6U MyFi Polymerase (Bioline) and 2 μl of purified initial amplicon. Thermocycling conditions were: 95°C 1 min, then 5 cycles 95°C 5 s, 55°C 10 s, 72°C 10 s. Products were purified as above and quantified by qPCR calibrated to known PhiX standards (Illumina) using the SYBR FAST qPCR Kit (Kapa Biosystems) on a RotorGene RG-6000 (Corbett).

Indexed libraries were pooled and 16 pM aliquots were paired-end sequenced on a MiSeq sequencer using a 600-cycle Version 3 kit (Illumina). The MiSeq Bcl output files were de-multiplexed and converted to FASTQ files using MiSeq Reporter v2.6 software (Illumina).

### Data analysis

FASTQ files were processed using a custom Python script to remove primer sequences, low-quality bases from the 3' end (phred < 30) and PCR artefacts. We demanded both the forward and reverse reads contribute to sequences passing filter. Singletons were excluded. Minimum combined sequence length after trimming was 280 bases and minimum contribution of individual trimmed reads was 30 bases. This generated our High Quality (HQ) data set.

We assessed the extent to which our reference DNA barcodes fully represented the sequences recovered from scats. For this we used FLASH [27], [parameters set:—m = 10, -x = 0.02, -M250]) to merge the *ndhJ* forward and reverse HQ reads for each sample. *RbcL* derived sequences did not merge since the amplicon size exceeded the length of the forward and reverse trimmed reads.

The five most abundant merged *ndhJ* sequences from each sample were used to create a non-redundant sequence set. This sequence set was subsequently compared to our *ndhJ* references by BLASTn [28]. Sequences that were not 100% identical to our references were considered novel and examined further. BLASTn searches were performed against NCBI’s non-redundant database and sequences that matched with three mismatches or less were annotated with the species name of the database entry. Equidistant matches, i.e. when a query sequence matched equally to two or more database sequences or no hits with less than four mismatches the query sequence, were deemed ‘Unknown’ with the sample identity in brackets. Thus, we extended our *ndhJ* reference set by 48 additional reference sequences.

Read mapping of the HQ data to the extended *ndhJ* and the *rbcL* references was performed by Bowtie 2 V2.2.6 [29] using the following settings:—end-to-end—score-min L,0,-0.045—mp 3—rdg 3,3—rfg 3,3—no-unal—very-sensitive—no-mixed—no-discordant—maxins 1000—reorder -k 500—mm. We post-filtered the BAM files to remove sequences that aligned with more than three mismatches and those that mapped to more than two references. We then summarized the read counts.

### Identification of dietary species

Trimmed and cleaned sequence reads were ranked according to frequency and plant species representation within each scat calculated as a proportion of the total. Species with a proportional representation below 0.1% were discarded as trace components. An arbitrary threshold of 1% of counts from a scat was imposed for plant species to be included for display purposes. ‘Unknown’ sequences were subject to a BLASTn search (https://blast.ncbi.nlm.nih.gov) and the top hit recorded.

### Multivariate analyses

Principal Component analyses (PCAs) were performed on normalised barcode counts (natural log transformation) recovered from all scats using the computer program MVSP (Multi-Variate Statistical Package version 3.22, Kovach Computing Services).

### Plant toxicological analysis

*Carrichtera annua* was analysed by a modified glucosinolate method [30]. Milled plants (40 mg) were heated (75°C, 5 min) to deactivate myrosinase, then extracted with aqueous methanol (2 mL, 70%, 70°C) for 20 min, centrifuged (5 min, 18°C, 6000 rpm) and supernatant collected. The residue was re-extracted twice, and the combined supernatant analysed on a Waters Acquity UPLC-PDA at 225 nm with an external calibration of Sinigrin (Sigma-Aldrich) for quantification. Glucosinolates were separated on a UPLC Acquity BEH C18 column (50 mm x 2.1 mm id, 1.7 μm) at 35°C, eluted with 0.1% formic acid in 10 mM ammonium acetate (A) and acetonitrile (B) at 0.2 ml/min and a linear gradient: 3% B to 60% B in 8 min. Glucosinolates were identified based on mass and reported fragment ions [31] on an UHPLC-MS QExactive (Thermo Fisher Scientific) equipped with a Dionex Ultimate 3000 UHPLC using identical chromatographic conditions as above. For each compound, a full scan in positive (ESI) ionization mode was acquired at a resolving power of 70,000 full width half maximum. A MS scan range *m/z* 300–1800 was selected, with a MS^2^ scan range *m/z* 50–500.

*Moraea setifolia* was screened for bufadienolide-type cardiac glycosides using an adapted method of [32] after reports suggesting their presence in this species [33]. Milled plants (100 mg) were mixed with 2 ml 95% ethanol containing 200 μl m-dinitrobenzol (10 μg/ml methanol) as internal standard and incubated at 78°C (1h). Samples were centrifuged (10 min, 18°C, 4500 rpm), and the supernatant collected. The extraction was repeated twice and combined supernatants evaporated to dryness under nitrogen at 78°C. The residue were re-dissolved in 1 ml methanol, and analysed according to [34], using UPLC system described above. A Waters Acquity HSS T3 column (100 mm x 2.1 mm id x 1.8 μm) was eluted at 0.250 ml/min with a mobile phase comprising 0.1% formic acid (A) and acetonitrile (B) and a linear gradient: 15% B to 50% B (25 min), 50% B to 45% B (5 min). Traces were monitored at 300 nm with bufalin (Sigma-Aldrich) as an external standard. The samples were also analysed on a UPHLC-MS/MS.

*Asphodelus fistulosus* was assessed for anthraquinones (potential photosensitizers) following reports of these compounds in this species [35]. Milled plant (5 g) was sequentially extracted with n-hexane, ethyl acetate and methanol.

*Rostraria cristata* is favored as a source of fodder in several parts of the world [36,37] and so was not subject to chemical analysis.

## Results

### Plant community structure

The three locations diverged in plant community structure (S1 Table). Moorunde was open woodland with a patchy canopy (*Eucalyptus gracilis*; *Eucalyptus oleosa* and *Myoporum platycarpum*) over scrub (*Geijera linearifolia*; *Senna artemisioides*; *Enchylaena tomentosa*; *Moraea setifolia; Zygophyllum* spp.; *Carrichtera annua* and *Brassica tournefortii*). Kooloola was a closed scrubland (*Atriplex stipitata*; *Maireana* spp.; *Marrubium vulgare*; *Ajuga iva*; *Sida corrugate*; *Moraea setifolia*; *Carrichtera annua* and *Brassica tournefortii*) with highly scattered *Eucalyptus oleosa*. Portee was more degraded, with closed herbland dominated by *Carrichtera annua*, *Asphodelus fistulosus* and *Moraea setifolia* over *Erodium crinitum*, *Carrichtera annua* and *Brassica tournefortii*. Moorunde contained most species (56; 25 site-specific) followed by Kooloola (37 species; 11 site-specific) and Portee (27 species; 2 site-specific). Overall, there were just sixteen plant species present in all three sites, of which the majority (12) were aliens (Table 1). Given the widespread but uneven nature of symptomatic wombats across the Murraylands, particularly during summer, we next sought to investigate whether these widely distributed alien species featured significantly in their summer diets.

**Table 1:** Plant species present in the three study sites in the Murraylands, South Australia.

Plant species present in Moorunde (M), Kooloola (K) and Portee (P)	
Native species	Alien species
*1. Austrostipa sp*. *2. Eucalyptus oleosa (M*,*K)* *3. Euphorbia drummondii* *4. Rhytidosperma caespitosa*	*5. Ajuga iva (M*,*K*,*P)* *6. Brassica tournefortii (M*,*K*,*P)* *7. Calotis hispidula* *8. Carrichtera annua (M*,*K*,*P)* *9. Erodium crinitum (M*,*K*,*P)* *10. Heliotropium europaeum* *11. Herniaria cinerea* *12. Hypochaeris radicata* *13. Moraea setifolia (M*,*K*,*P)* *14. Rostraria cristata* *15. Silene apetala* *16. Sonchus oleraceus*

Species that were dominant in the plant communities of Moorunde (M), Kooloola (K) and Portee (P) are indicated in brackets.

### Plant barcode reference library

Construction of a local reference barcode library is the first step in dietary reconstruction by metabarcoding. Our reference library (114 specimens) included 83 samples morphologically identified to species, six to genus and three unknowns. DNA barcodes were recovered from all species and genera for *rbcL*, and all but 4 species for *ndhJ* (*Amyema miquellii*, *A*. *preissii*, *Goodenia pinnatifida*, *Zygophyllum apiculatum*). The 3 unknowns were assigned to two tribes (Camphorosmeae, Amaranthaceae and Gnaphalieae, Asteraceae) following BLASTn alignment. UPGMA trees of these sequences revealed 71 unique barcodes for *ndhJ* and 78 for *rbcL* (S1 Fig).

### Diet reconstruction from autopsy samples

**Fig 1:**
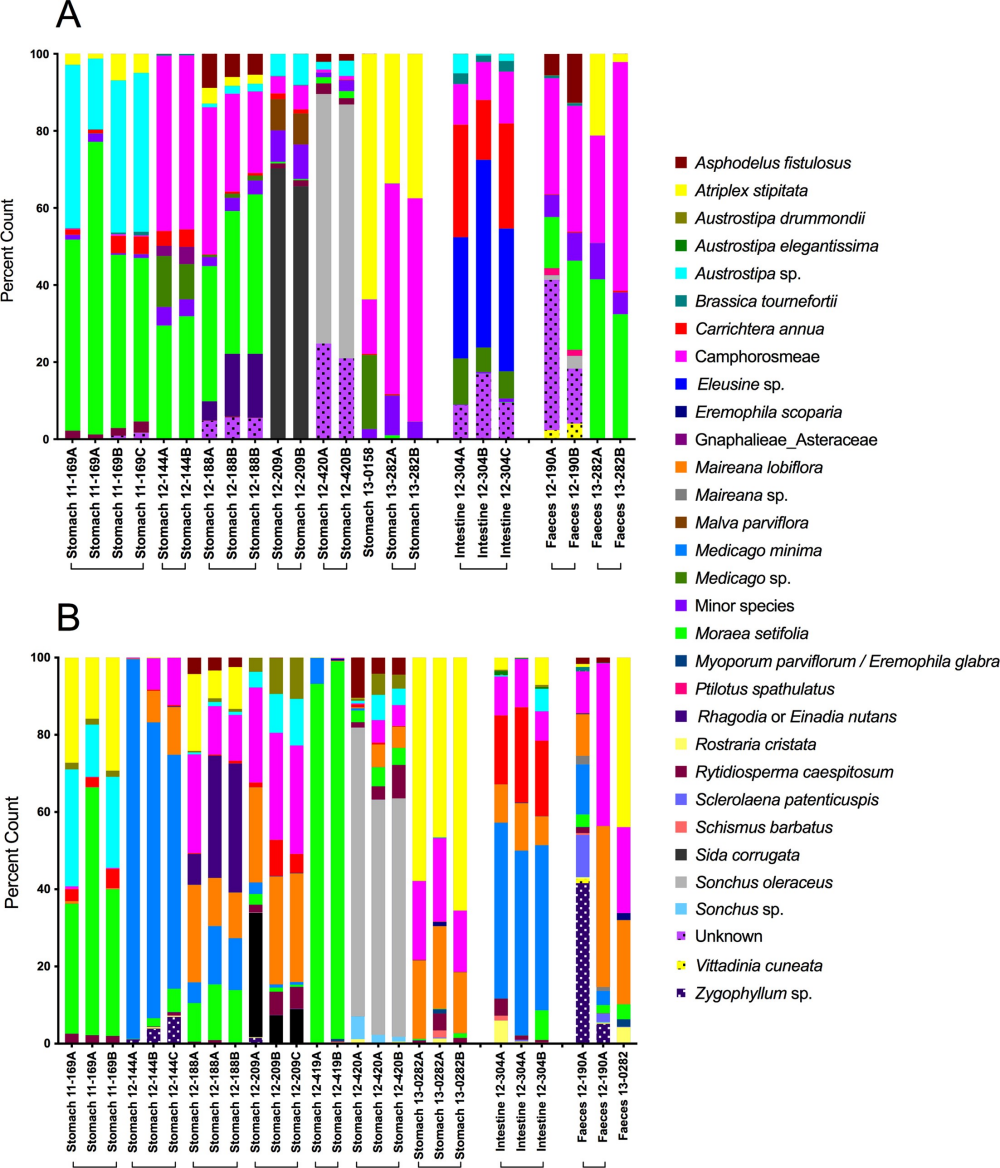
Diets of deceased southern hairy nosed wombats. Diets reconstructed from three parts of the alimentary canal (stomach, long intestine and fecal [scat] samples) using *ndhJ* (panel A) and *rbcL* (panel B). Histograms represent percentage of *ndhJ* or *rbcL* barcodes matching one of the species shown in the margin. Codes on x-axis indicate the organ used, a numeric indication of year (12 = 2012), a numeric identifier of the animal. The final letter (A-C) indicates sample replicates, with technical replicates possessing identical letters.

*NdhJ* and *rbcL* metabarcode profiles were constructed from alimentary canal samples of ten autopsied wombats. Sequence recovery was far higher for *ndhJ* than *rbcL*. We uniquely mapped 1,316,225 *ndhJ* sequences to a single taxonomic group (mean: 48,749 per sample), compared with 90,817 for *rbcL* (mean 3,493 per sample). Diets reconstructed from biological and technical replicates of the same animal and taken from the same part of the alimentary tract were similar for both markers (Fig 1). There was also broad congruence between diets reconstructed from the two markers, despite substantial variation between animals (Fig 1).

*RbcL* profiles contained 14 times fewer unique matches than *ndhJ* and were slightly more variable between replicates. There were nine instances where a species was present only in one *rbcL* replicate diet. This compares with four instances for *ndhJ*. Lower absolute counts recovered for *rbcL* were also associated with wider variation between replicates in percentage estimates of species composition. However, the lower sequence yields of *rbcL* were partly compensated by an increased ability to distinguish between taxa. *RbcL* alone was able to separate *Maireana lobifolia* from the Camphorosmeae group (Amaranthaceae), and also *Austrostipa elegantissima* and *A*. *drummondii* from other *Austrostipa* species. Overall, the profiles from the autopsy replicate samples indicate that *ndhJ* provides far higher sequence yields and so creates more stable estimates of dietary composition than *rbcL*, despite the latter’s slightly increased capacity to distinguish between some taxa.

**Fig 2:**
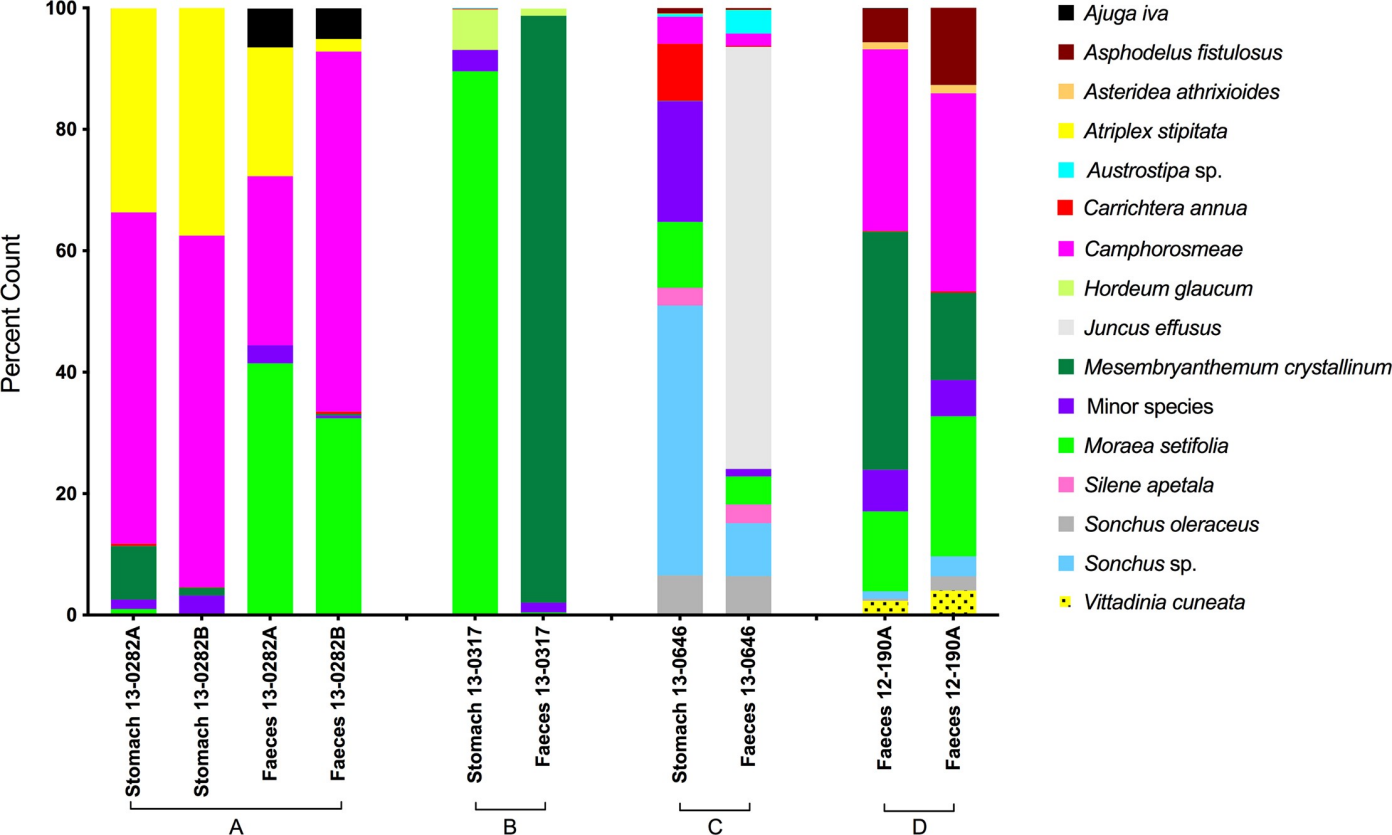
Within-animal variation in reconstructed diets of deceased southern hairy nosed wombats. Diets reconstructed using *ndhJ* sequences recovered from replicated stomach and fecal samples of three animals (A-C), with one animal yielding only fecal samples (D). Histograms represent percentage of reference *ndhJ* barcodes matching one of the species shown in the panel for: (A) stomach and fecal profiles from animal 282; (B) stomach and fecal profiles from animal 317; (C) stomach and fecal profiles from animal 646 and D technical replicates of a fecal profiles of animal 190.

In three cases, we were able to compare *ndhJ* dietary profiles from stomach and anal scats of the same individual. There were inconsistencies in every case. In animal 13–0282, the replicated stomach diets (Fig 2A) were similar to those of the scats but lacked *Ajuga iva* as a minor component. The stomach and scat profiles of animal 13–0317 were widely divergent, with *Hordeum glaucum* the only species present in both profiles (Fig 2B). The stomach profiles of animal 13–0646 lacked *Juncus effusus* which was abundant in scat profiles (Fig 2C). A fourth animal yielded only a single useable scat sample but the dietary profiles of the two technical replicates from this animal were very similar (Fig 2D).

Incongruence between stomach and anal scat profiles could plausibly arise from episodic feeding, intra-scat heterogeneity or technical inconsistencies. We recovered between 14,447 and 55,848 sequences from half-scat replicates and uniquely mapped 85% - 91% of input reads. Moreover, twinned samples varied in the number of absolute counts retrieved from each sample (by 2–37%) but the resultant profiles invariably shared the same species identity and rank order for main dietary components, with only modest variation in proportions (S2 Table). The identity of most minor dietary components was also conserved, although there were some differences between pairs in the minor species visible above the 1% (display) threshold. However, the 'absent' species were invariably present in the replicate half but at levels between 0.1% and 1% thresholds and so may have been a consequence of the variation in total sequence reads. Thus, only modest variance seems attributable to technical factors or intra-scat heterogeneity. We therefore infer that wide divergence observed between stomach and scat profiles is most consistent with episodic feeding.

### Diet of captive wombats

**Fig 3:**
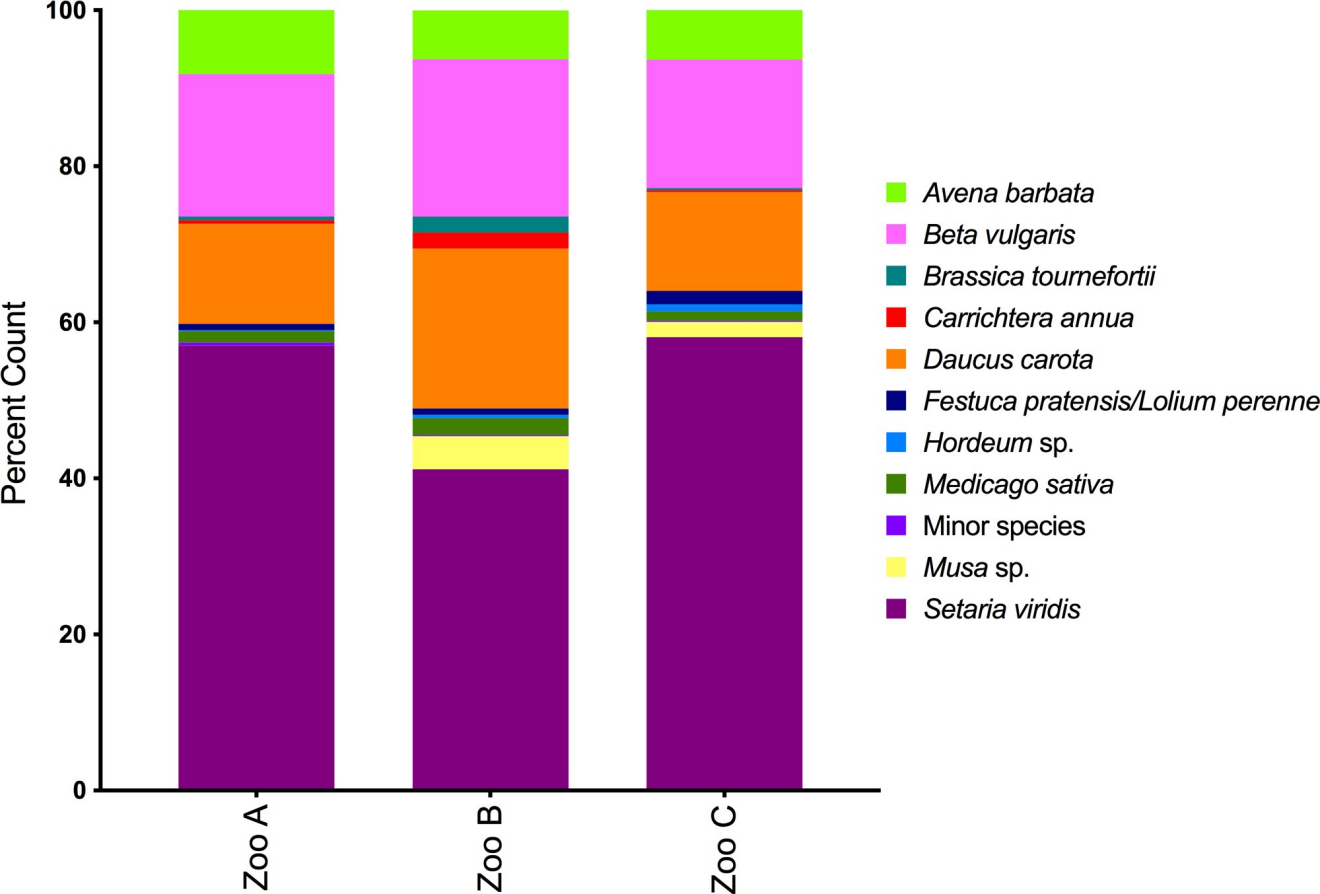
Diets of captive southern hairy nosed wombats. Diets reconstructed using *ndhJ* sequences from feces of three zoo animals (Zoo A-C) provided a semi-controlled diet. Histograms represent percentage of reference *ndhJ* barcodes matching species shown in the panel.

We next reconstructed *ndhJ* profiles using scats from three captive zoo animals for which living environment and diet was essentially the same. The *ndhJ* diets of these three animals shared the same major and most minor component species (Fig 3). These were congruent with partly characterized diets of these animals (S1 Text), which were dominated by cereals, commercial grasses, vegetables, and both native and alien plants in their enclosure (including trace amounts of *Carrichtera annua*).

### Diet of wild wombats

We mapped 86% (6,116,612/7,130,787) of clean *ndhJ* reads to a unique reference barcode and 3% (223,789/7,130,787) to two reference barcodes from the NCBI database. Overall, *ndhJ* mapping frequencies for individual scats exceeded 90% (mean 92%). Success rates were far lower for the longer *rbcL* locus, where we recovered 1,244,055 clean sequences across all scats, with 797,480 (64%) mapped uniquely to a local reference barcode and 82,826 (7%) to two reference (NCBI) barcodes. The residual 29% failed to map to any reference barcode. The percentage of sequence reads that mapped to reference barcodes varied considerably between individual scats and was lower than *ndhJ* (mean 73.5%). The superior performance of *ndhJ* led to its use as the primary source of information for dietary reconstruction for comparison between sites.

**Fig 4:**
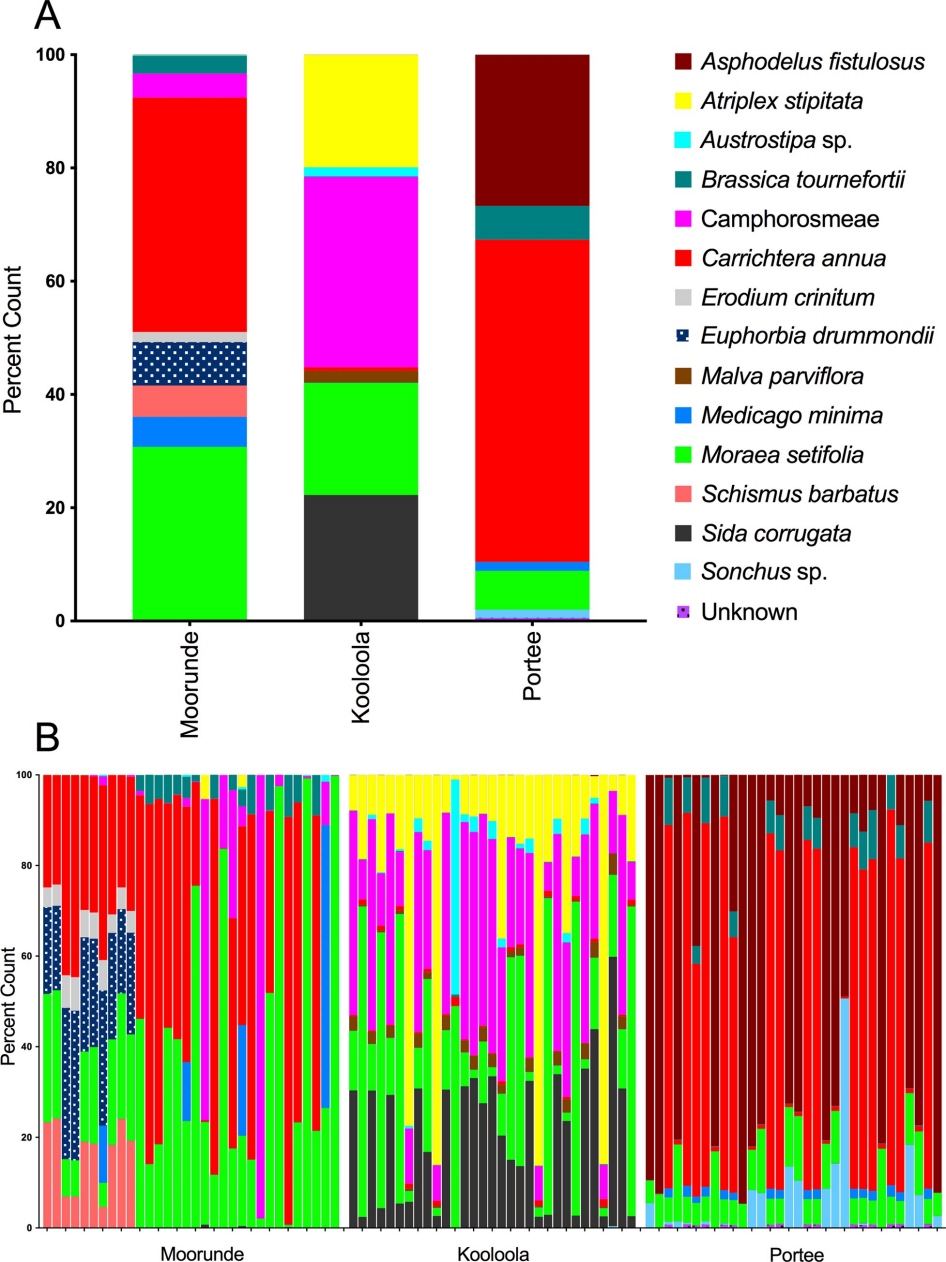
Summer diets of southern hairy nosed wombats in the Murraylands reconstructed from *ndhJ* sequences. Panel A shows population-wide diets reconstructed using *ndhJ* sequences from all samples in Moorunde, Kooloola and Portee sites. Panel B shows diets of individual samples collected in each of the three sites. Diets are represented as the percentage of sequences matching to one of the species shown in the key.

Population-averaged **Moorunde** scat *ndhJ* profiles suggested wombats in this location had the most diverse summer diet (Fig 4A), featuring *Carrichtera annua* (41%) and *Moraea setifolia* (30%) prominently, and a significant presence of *Euphobia drumondii (8%)*, *Medicago minima* (5%), *Schismus barbatus* (5%), unknown Camphorosmeae (Amaranthaceae) (4%), *Brassica tournefortii* (3%) and *Erodium crinitum* (2%). Thus, the diet is dominated by the same two plant species that dominate the ground canopy (*Carrichtera annua* and *Moraea setifolia;* S1 Table). The near absence of the native grass genus *Austrostipa* (0.14%) from both ground cover and reconstructed diet is also worthy of note. There was considerable intra-site variability between scats, although this was minor compared to inter-site variation (Fig 4B). Most individual Moorunde scats followed a similar pattern to the population as a whole, with either *Carrichtera annua* or *Moraea setifolia* dominating in all but three scats (Fig 4B). Two dietary components (Camphorosmeae, Amaranthaceae and *Medicago minima*) were abundant/dominant in only a few scats whereas most other components (e.g. *Brassica tournefortii*) appeared commonly in modest amounts across many scats.

Population-averaged **Kooloola** diet contained four main components: *Sida corrugata* (22%); *Atriplex stiptata* (19%); *Moraea setifolia* (19%) and the Camphorosmeae group (Amaranthaceae) (32%) (Fig 4A). *Malva parviflora*, *Carrichtera annua*, and *Austrostipa* sp. were significant minor dietary components (1–2%). Thus, two of the dominant ground cover species (*Atriplex stiptata* and *Moraea setifolia*) featured prominently, with the remaining main dietary components (*Sida corrugata* and Camphorosmeae, Amaranthaceae) both common in the ground flora (S1 Table). Diets from individual scats were more consistent in Kooloola than elsewhere (Fig 4B) and featured the same four dominant species groups seen in the averaged diet, although relative proportions varied. *Moraea setifolia* and *Atriplex stiptata* were most variable (4–86% and 2–69% of total diet, respectively) whereas *Sida corrugata* and the Camphorosmeae (Amaranthaceae) group were more consistent (2–41% and 6–47%, respectively; Fig 4). Of the minor dietary components only *Malva parviflora* appeared in more than half of the samples (20/29), and none reached 4% of the profile for any scat (Fig 4B).

Population-averaged **Portee** site diet was notable by domination of *Carrichtera annua* (57%) and *Asphodelus fistulosus* (27%) (Fig 4A). *Moraea setifolia* (7%), *Brassica tournefortii* (6%), *Medicago minima* (2%) and *Sonchus* sp. (1%) formed significant minor components. The two main dietary species (*Carrichtera annua* and *Asphodelus fistulosus*) again matched the dominant ground cover for the site (S1 Table). Despite the relative simplicity of the pooled diet, there was extensive variation between individual scats, with no species universally present (Fig 4B). Minor species were similarly variable in presence and abundance (Fig 4B).

### Diet comparison between sites

There was a clear relationship between the major dietary components and the species composition of the local plant community. Moreover, the diets of wombats overwhelmingly featured those plant species that also dominated the plant communities from which they were taken (S3 Table). Indeed, plant species found to dominate the canopy, understorey or ground cover of these communities accounted for 88%, 99% and 99% of the reconstructed diets of wombats from Moorunde, Kooloola and Portee respectively (S3 Table). There was nevertheless clear evidence of the selective feeding. Over half of the dominant species in Moorunde and Portee failed to feature in the diets of any individuals and three dominant species were absent from the reconstructed diets from Kooloola (S3 Table). Equally, two relatively infrequent species from Moorunde (*Euphorbia drummondii* and *Medicago minima*) and one from Kooloola (*Sonchus* sp.) featured in the reconstructed diets at levels far above their ground cover presence (S3 Table).

**Fig 5:**
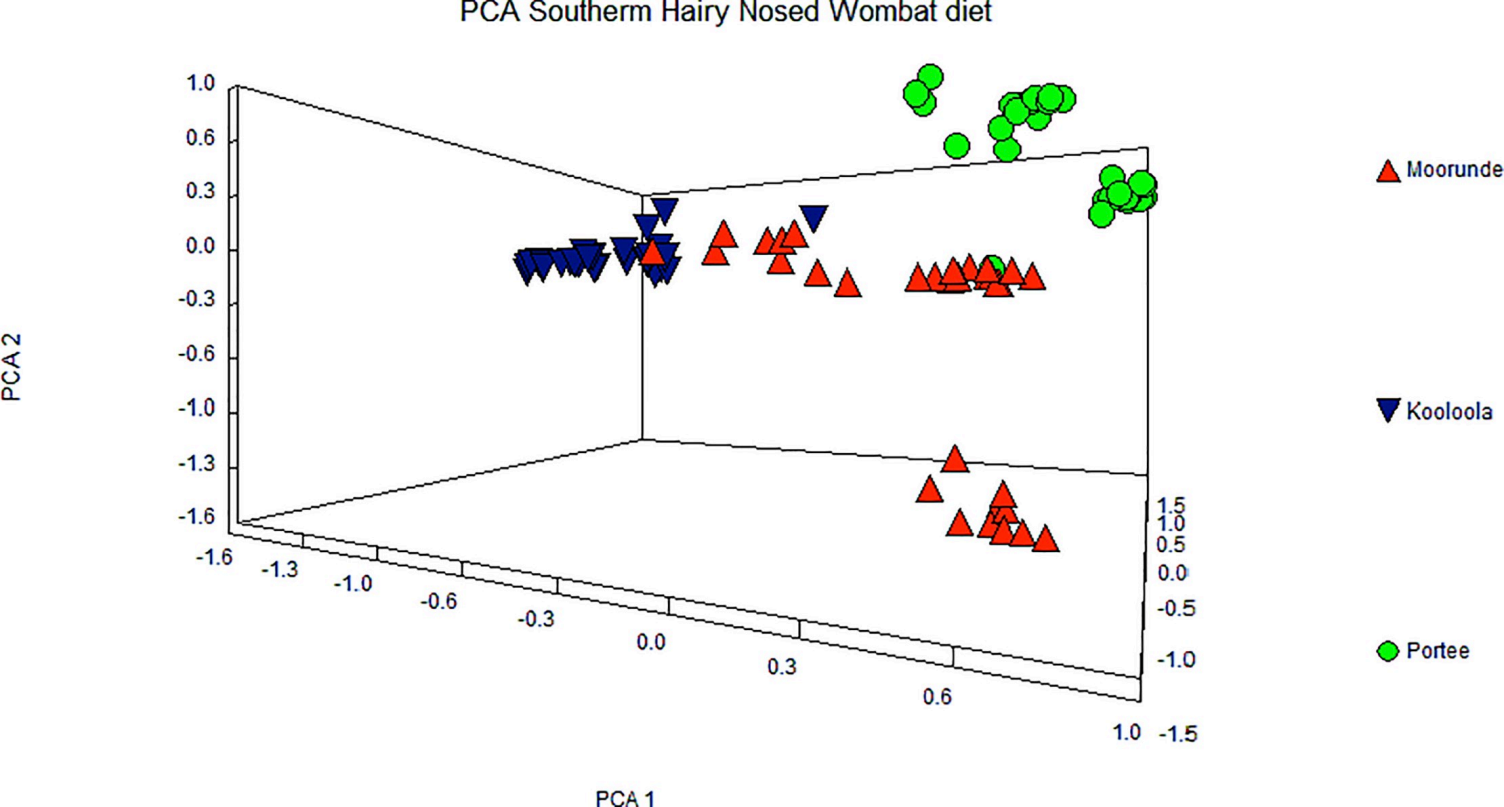
Principal Component Analysis (PCA) of reconstructed diets from three locations. PCA of *ndhJ* sequences from fresh feces of southern hairy nosed wombats collected in: Moorunde (red triangles); Kooloola (inverted blue triangles) and Portee (green circles) in the Murraylands, South Australia. The three axes represent 80% of total variation.

A 3D multivariate PCA plot of individual scat profiles from all sites and representing 80% of total variation revealed evidence of structuring between locations, and revealed clear separation of scats from Kooloola and Portee (Fig 5). However, a small number of individual scats from the most floristically-diverse Moorunde site co-clustered with scat profiles more typical of both Kooloola and Portee (Fig 5).

### *RbcL* diets

**Fig 6:**
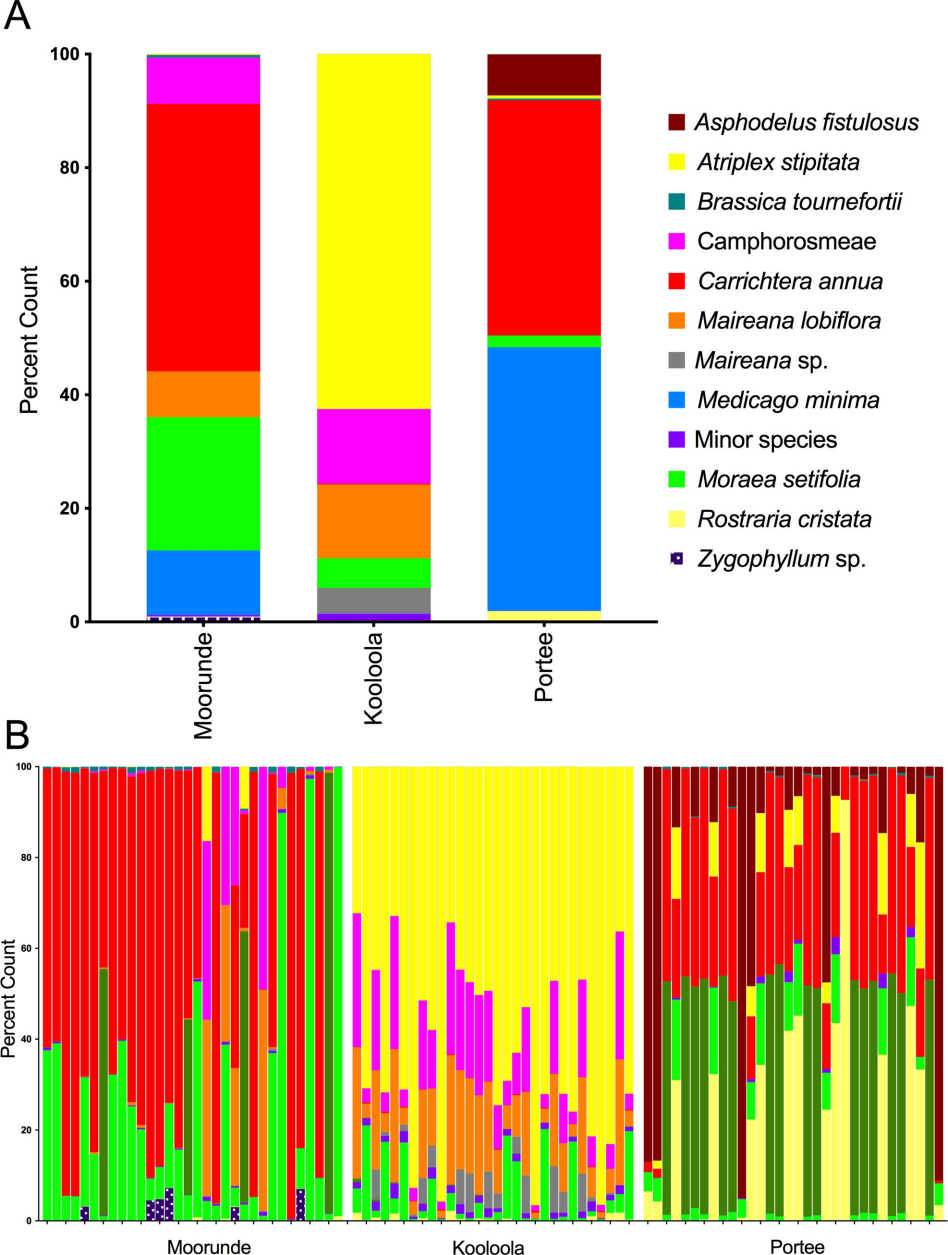
Summer diets of southern hairy nosed wombats in the Murraylands reconstructed using *RbcL* sequences. Diets reconstructed using *RbcL* sequences recovered from southern hairy nosed wombat scats. Panel A shows the population-wide diets reconstructed using *rbcL* sequences from all samples collected in Moorunde, Kooloola and Portee sites. Panel B shows the diets of individual samples collected in each of the three sites. Diets are represented as the percentage of sequences matching to one of the species shown in the key.

The *rbcL* diets were broadly congruent with *ndhJ* diets but were more variable and based on fewer sequences (Fig 6). However, several minor components from the *ndhJ* profiles were consistently absent from *rbcL* profiles. *Euphorbia drumondii*, *Schismus barbatus*, *Brassica tournefortii* and *Erodium crinitum* were missing from Moorunde, *Sida corrugata* from Kooloola and *Brassica tournefortii* and an unresolved *Sonchus* species from Portee (Fig 6). Thus, despite having increased ability to separate taxonomic groups, *rbcL* profiles were more volatile and generally featured fewer components.

### Clinical and pathological investigations

All seven clinically examined wombats from Portee were in poor to emaciated body condition, with moderate to extensive alopecia, poor hair quality and discoloration, and with dermatitis present in six animals. Haematological and biochemical analysis showed a poorly regenerative anemia (n = 5) and hypoproteinaemia characterized by hypoalbuminemia (n = 5), and low creatinine (n = 5) consistent with malnutrition, chronic disease, and/or toxicity. In four individuals, moderate elevations in serum ALP, AST, ALT, GLDH and/or GGT activities and bilirubin was suggestive of hepatic disease. A severe hepatopathy was confirmed in one of these individuals at autopsy (12–0399), although autopsies were not performed on the remaining three.

### Autopsy investigations

Condition of wombats varied widely between sites (S4 Table). All wombats from Portee were in poor to emaciated body condition and showed moderate to extensive alopecia and hair coat discoloration. Three showed severe hepatopathy (megalocytosis, hepatocellular necrosis, bile duct hyperplasia, cholestasis, bridging fibrosis), two of which also had photosensitive dermatitis. Three further animals exhibited milder non-specific hepatic lesions (bile duct hyperplasia, periportal to bridging fibrosis, hepatocellular anisokaryosis) and one had hepatic atrophy. The four wombats examined from Moorunde were in poor to moderate body condition. All had dermatitis with intralesional mites but only one showed mild hepatic lesions (mild biliary hyperplasia and periportal fibrosis). Two ‘road kill’ wombats from Kooloola were in good physical health prior to death. Major pathological findings from seven additional Murraylands wombats from outside the study sites indicated a similarly patchy occurrence of hepatic disorders in the region (S4 Table).

### Plant toxicology

We first sought to identify alien species most likely to affect the health of wombats exhibiting symptoms of ill-health. Four of the twelve alien species found in all sites failed to appear in the reconstructed diets of any animal above 1% (viz: *Calotis hispidula; Heliotropium europaeum; Herniaria cinerea*; *Hypochaeris radicata*) (S2 Table, Figs 1, 2 and 4) and so were discarded from further analysis. Only four of the remaining eight species (*Brassica tournefortii*, *Carrichtera annua*, *Moraea setifolia* and *Rostraria cristata*) featured in the diets of deceased wombats exhibiting symptoms at the time of death (Fig 1; S4 Table). The last of these (*Rostraria cristata*) is favored as a source of fodder in several parts of the world (see above) and so was discarded from toxicological analysis. Of the remaining three alien species examined, no known toxic principles were detected in *Moraea setifolia* (specifically, no bufadienolides) or *Asphodelus fistulosus* (no anthraquinones). However, high levels of glucosinolates were recovered from adult *Carrichtera annua* (128–149 μmol/g DW). Far lower levels were recovered from dry dead material and seeds (2–21 μmol/g DW) of this species (S5 Table).

## Discussion

There has been an impressive expansion in the number and diversity of plant reference DNA barcodes available for use in dietary studies [20]. However, the core plant barcodes (*rbcL* and *matK*) have residual limitations for use in herbivore diet reconstruction. Poor amplification of *matK* in many plant species [19,20] and the modest information content of *rbcL*, when coupled with inherent difficulty in securing high quality DNA sequence from fecal samples has led to most groups to favor more variable and shorter loci such as *trnL* [17,21], despite the need to create study-specific reference barcodes. Even in the presence of such precautions, however, no metabarcoding protocol is free from error.

Biological replicates in the present study exhibited up to 40% variation in the total number of barcode sequences that passed quality filter. Insufficient barcode read recovery in a profile has potential to increase error in percent estimations of species abundance and may markedly skew estimates of minor species components (rare barcodes being missed or over-represented). Such differences in total count recovery may be readily explained by modest compositional differences between replicate scat halves or minor variation in technical processes such as library preparation. Despite such differences, broad consistency between the percent species composition of technical (PCR) and biological replicates provides some support the tenet that the barcode profiles do provide a reasonable indication of the species mix in the scats, particularly for the major dietary component species.

Of far greater concern is the possibility of systematic errors. The propensity of all barcode loci to fail or only weakly amplify some taxonomic groups [38] creates scope for the omission of some dietary components or else for the systematic under-representation of inefficiently amplified species. Comparison between diets reconstructed using different loci helps uncover such discrepancies. The two markers used here differ in length and the species they reliably amplify [24]. However, both separated the vast majority of plant species and generated broadly congruent diet profiles. Despite its increased information content, *rbcL* produced simpler profiles and omitted some of the minor dietary components detected by *ndhJ*. Accordingly, the latter was deemed most reliable marker for dietary reconstruction. Further support for this stance came from the close match between the partly characterized (and very distinct) diets of captive animals and their scat profiles. Equally, the broad congruence between the ground cover species at all three wild sites and the population-wide scat profiles adds further support that such profiles probably provide at least a crude approximation of the ingested diet.

Care is nevertheless needed when relating scat profiles to biomass ingested by animals. Plant species and tissues vary in the number of chloroplasts or proplastids per cell/tissue [39] and genomes per plastid (see [40]). Barcode count frequency from digested or semi-digested samples therefore can only be viewed as providing an indicative guide of major and minor dietary components, and not a truly quantitative reflection of the diet (without extensive calibration). Biological sources of error could also distort dietary profiles. For example, if the herbivore exhibits episodic feeding, then wide variances may appear between scat profiles. Indeed, our results provided some evidence of episodic feeding in two autopsied animals. Tellingly, *Juncus effusus* was a dominant component in scat profiles of one animal, *Mesembryanthemum* in another but both species were missing from the corresponding stomach profiles. Absence from the stomach discounts the possibility of preferential digestion and when considered together with the high abundance and distinctness of both dominant barcodes, strongly suggests episodic feeding. Episodic feeding may offer an additional explanation for some of the inter-sample variation seen in all populations. Despite clear separation between scat profiles from the three locations, subclustering within each site is again suggestive that episodic feeding is commonplace.

Overall, summer diets of the SHNWs seem heavily determined by location; a feature that is perhaps unsurprising for a territorial, burrowing herbivore whose feeding options are highly limited during dry summer months. The main dietary species at all sites closely matched the ground coverage, suggesting that the SHNWs continue to feed in summer and display limited levels of selection during this stressful period.

Invasive plants can alter native plant community structure and reduce availability of ‘safe’ food for herbivores [41]. For resident herbivores with seasonal food shortages, community shifts can compel consumption of potentially toxic plants to avoid starvation [42]. There were five alien species that were abundant in all sites. Of these, glucosinolates have been reported in *Brassica tournefortii* [43] and were found here in high quantities in *Carrichtera annua*. We found no evidence of any toxic principles in the other three species. *Brassica tournefortii* and *Carrichtera annua* featured in all sites but were far more prominent in scats from Portee; accounting for more than half of the reconstructed diet. For one animal, these were the only dietary species observed in the scat and in four others, they exceeded 80%. Excessive intake of glucosinolates has been associated with several health problems in livestock, notably including reduced feed intake and growth, hepatic lesions (bile duct hyperplasia, megalocytosis, zonal hepatocyte necrosis, and hepatic fibrosis), photosensitivity and anemia [44,45]. All wombats examined from Portee showed signs of severe weight loss, alopecia and changes in hair color and quality. Blood testing (n = 7) also suggested anemia, hypoproteinaemia (hypoalbuminemia), and reduced creatinine consistent with malnutrition, chronic disease and/or toxicity, and in four individuals, elevations in serum ALP, AST, ALT, GLDH and/or GGT activities suggested underlying hepatic disease. Moreover, post-mortem examination of six of seven Portee wombats showed hepatic lesions consistent with glucosinolate poisoning. By comparison, scats from Kooloola contained <3% of the glucosinolate-containing aliens (*Brassica tournefortii* and *Carrichtera annua*) and the two animals examined from this site lacked signs of glucosinolate toxicity. The small proportion that did (6%; 5/81) may be plausibly explained by episodic feeding on these alien plants. It is also important to recognize the possibility of cumulative toxic exposure to other plants outside the summer period. The small number of Moorunde wombats examined lacked obvious signs of poisoning except for low body weight and mild non-specific hepatic lesions in one adult female. *Brassica tournefortii* and *Carrichtera annua* featured significantly in the diets of these animals but at a much lower level than seen in Portee.

In this study, we have focused on the impact of toxic alien invasive plants on the health of the wombats. However, we cannot discount the possibility that food shortage during summer led some animals to increase consumption of very toxic native plants. For example, members of the genus *Euphorbia* are widely reported to be toxic to mammals [46,47] but also appeared in the diets of some individuals featured in the current study. However, *Euphorbia drumondii* featured prominently in the diets of more than a quarter of wombats from Moorunde (where symptomatic animals are rarely observed) and was absent from the diets of any wombat from Portee (where symptomatic individuals are most common). It was also present in significant quantities in the stomach of an asymptomatic deceased wombat recovered from Moorunde (12–144) but absent from the diets of almost all deceased but symptomatic wombats. Nevertheless, the possibility remains that some animal may suffer from the effects of ingesting extremely toxic plants in small doses. This is something that requires further investigation.

## Conclusions

The most plausible explanation of the poor health of Portee wombats resides at least partly on their heavier reliance on glucosinolate-containing alien plants during summer. The same alien plants do feature in the summer diets of wombats in the more floristically-rich Kooloola and Moorunde. In these sites, it is tempting to postulate that there is sufficient dietary choice for most animals to manage their glucosinolate intake via total avoidance or episodic feeding on these plants. Excessive glucosinolate intake can potentially further exacerbate the recent population pressures on SHNWs particularly if habitat degradation such as that seen in Portee becomes more widespread. To reverse these trends, we advocate the prioritised control of *Carrichtera annua* and *Brassica tournefortii* as part of habitat restoration programs. Supplementary feeding (particularly fresh grasses or cereals) during summer droughts may also provide support for the affected populations while such restorations take effect.

More generally, we note the efficacy of episodic feeding as a defense against poisoning is inextricably linked to the diversity and abundance of other food plants available. We therefore argue that protection against population decline in any territorial herbivore by poisoning requires knowledge of all components of the diet, not just those that contain toxic principles.

## Supporting information

S1 Text Diet provided to captive southern hairy nosed wombats. (DOCX)

S1 Table Plant community descriptions and plant species present in three sites (Moorunde; Kooloola and Portee) within the Murraylands of South Australia. (DOCX)

S2 Table Table showing the number of unique species *ndhJ* matches in biological replicates taken from four fresh feces collected in Moorunde. The biological replicates were taken from alternate halves of each scat. Each table shows all mapped sequences.
(XLSX)

S3 Table Table comparing the abundance of species in the plant communities of Moorunde, Kooloola and Portee (South Australia) with the estimated percentage presence in the diet reconstructed by *ndhJ* metabarcoding. (XLSX)

S4 Table Clinical and pathological findings of post-mortum examinations of Southern Hairy Nosed Wombats (SHNW) recovered from the Murraylands region of South Australia. (DOCX)

S5 Table Glucosinolate content of *Carrichtera annua* green plants, seedlings and dead material/seed debris collected at each of the three study sites. (DOCX)

S1 Fig UPGMA trees of reference barcodes. Unrooted UPGMA trees of reference DNA barcode sequences of plant species collected from three sites in the Murraylands of South Australia (Moorunde, Kooloola, Portee) generated using Geneious 8.0 (www.geneious.com, Kearse et al. 2012. Bioinformatics **28**:1647–1649). Panel A represents separation of *ndh*J sequences. Panel B represents separation of *rbc*L sequences.
(PDF)
