# Supplementary material for: Dual-locus DNA metabarcoding reveals southern hairy-nosed wombats (Lasiorhinus latifrons Owen) have a summer diet dominated by toxic invasive plants
Source: PLoS One. 2020 Mar 6;15(3):e0229390. doi: 10.1371/journal.pone.0229390 (PMC7059939; doi:10.1371/journal.pone.0229390)
Supplement: S1 Text — (DOCX) [file pone.0229390.s001.docx]

**S1 Text.** Diet provided to captive Southern Hairy Nosed Wombats

Relative quantities of the following dietary components were subject to availability and

not strictly controlled. However, the species composition of the feeds were as follows:

**Morning feed:**

Root vegetables: *Daucus carota* (carrots), *Pastinaca sativa* (parsnips), *Brassica rapa* (turnips), *Brassica napus* (swedes)

Grains: capra goat mix (*Avena sativa*, *Hordeum vulgare*, *Zea Mays*, *Pisum sativum*, *Medicago sativa*, *Helianthus annuus*, *Glycine max, Sorghum bicolor)*

Leafy vegetables: *Brassica oleracea*, *Brassica napus*, *Brassica napus*

*Beta vulgaris, Lactuca sativa*

**Exhibit feed:**

*Avena sativa* hay, and cut grass (mix including *Festuca pratensis*, *Lolium perenne*, *Cynodon dactylon, Austrostipa* spp. *Setaria* spp.).

Occasionally some *Glycine max* and scatter of macropod pellets, thistles (*Cirsium vulgare*, *C. arvense*, *Carduus* spp. *Carthamus* spp.) when available.

Sequences downloaded from NCBI and added to the reference barcode dataset for scat analysis (NA – not available).

| **Species** | ***rbcL*** | ***ndhJ*** |
| --- | --- | --- |
| *Avena sativa* | *✓* | NA |
| *Beta vulgaris* | *✓* | *✓* |
| *Brassica napus* | *✓* | *✓* |
| *Brassica oleracea* | *✓* | *✓* |
| *Brassica rapa* | *✓* | *✓* |
| *Carduus nutans* | NA | NA |
| *Carduus pynocephalus* | NA | NA |
| *Carduus tenuiflorus* | *✓* | NA |
| *Carthamus lanatus* | NA | NA |
| *Carthamus tinctorius* | *✓* | NA |
| *Cirsium arvense* | NA | NA |
| *Cirsium vulgare* | *✓* | NA |
| *Daucus carota* | *✓* | *✓* |
| *Festuca pratensis* | *✓* | *✓* |
| *Hordeum vulgare* | *✓* | *✓* |
| *Lactuca sativa* | *✓* | *✓* |
| *Lolium perenne* | *✓* | *✓* |
| *Medicago sativa* | *✓* | NA |
| *Medicago truncatula* | *✓* | *✓* |
| *Pastinaca sativa* | *✓* | NA |
| *Setaria viridis* | *✓* | *✓* |
| *Sorghum bicolor* | *✓* | *✓* |
| *Spinacia oleracea* | *✓* | *✓* |
| *Taraxacum officinale* | *✓* | NA |
| *Triticum aestivum* | *✓* | *✓* |
| *Zea mays* | *✓* | *✓* |
