# Supplementary material for: Dual-locus DNA metabarcoding reveals southern hairy-nosed wombats (Lasiorhinus latifrons Owen) have a summer diet dominated by toxic invasive plants
Source: PLoS One. 2020 Mar 6;15(3):e0229390. doi: 10.1371/journal.pone.0229390 (PMC7059939; doi:10.1371/journal.pone.0229390)
Supplement: S1 Table — (DOCX) [file pone.0229390.s002.docx]

**S1 Table. Plant community descriptions and plant species present in three sites (Moorunde; Kooloola and Portee) within the Murraylands of South Australia.**

Plant community descriptions

Moorunde: Open shrubland/woodland over *Carrichtera annua*, *Moraea setifolia* and *Austrostipa* spp. *Carrichtera annua* is the predominant exotic annual in the groundstorey layer throughout the year, associated with occasional high coverage of *Brassica tournefortii* that appear and persist for long periods after rains.

Kooloola: Closed low shrubland dominated in summer by *Atriplex stipitata*, *Marrubium vulgare* and *Maireana* spp. Again, *Carrichtera annua* is the predominant exotic annual in the groundstorey layer throughout the year, associated with occasional high coverage of *Brassica tournefortii* that appear and persist for long periods after rains.

Portee: Closed herbland dominated in summer by *Asphodelus fistulosus*, *Carrichtera annua* and *Austrostipa* spp. *Carrichtera annua* is the predominant exotic annual in the groundstorey layer throughout the year, associated with occasional high coverage of *Brassica tournefortii* that appear and persist for long periods after rains.

*List and nature of the species found in each of these communities is given below.*

| **MOORUNDE** |  |  |  |  |  |  |  |
| --- | --- | --- | --- | --- | --- | --- | --- |
| **Species** | **annual (a)**  **perennial (p)** | **native (N)**  **exotic (E)** | **canopy dominant (c )** | **understorey**  **dominant (u)** | **groundstorey dominant (g)** | **Scattered**  **non-dominant (nd)** | **higher frequency season dependent (s)** |
| *Acacia nyssophylla* | p | N |  |  |  | nd |  |
| *Ajuga iva* | p | E |  |  | g |  | s |
| *Alectryon oleifolius* | p | N |  |  |  | nd |  |
| *Amyema miquelii* | p | N |  |  |  | nd |  |
| *Asteridea athrixioides* | a | N |  |  |  |  | s |
| *Austrostipa drummondii* | p (grass) | N |  |  |  | nd | s |
| *Austrostipa elegantissima* | p (grass) | N |  |  |  | nd | s |
| *Austrostipa hemipogon* | p (grass) | N |  |  |  | nd | s |
| *Austrostipa puberula* | p (grass) | N |  |  |  | nd | s |
| *Austrostipa scabra falcata* or *A. nitida* | p (grass) | N |  |  |  | nd | s |
| *Brachyscome ciliaris* | a - p | N |  |  |  | nd | s |
| *Brassica tournefortii* | a - p | E |  |  | g |  |  |
| *Bupleurum semicompositum* | a | E |  |  |  |  |  |
| *Calotis hispidula* | a | E |  |  |  |  |  |
| *Carrichtera annua* | a | E |  |  | g |  |  |
| *Crassula colorata* | a | N |  |  |  |  |  |
| *Daucus glochidiatus* | a | N |  |  |  |  |  |
| *Dissocarpus paradoxus* | a - p | N |  |  |  | nd | s |
| *Enchylaena tomentosa* | p | N |  | u |  |  |  |
| *Eriochiton sclerolaenoides* | p | N |  |  |  | nd |  |
| *Erodium crinitum* | a - p | E |  |  | g |  | s |
| *Eucalyptus gracilis* | p | N | c |  |  |  |  |
| *Eucalyptus oleosa* | p | N | c |  |  |  |  |
| *Euphorbia drummondii* | a | N |  |  |  |  | s |
| *Geijera linearifolia* | p | N |  | u |  |  |  |
| *Goodenia pusilliflora* | a - p | N |  |  |  | nd |  |
| *Heliotropium europaeum* | a | E |  |  |  |  | s |
| *Herniaria cinerea* | a | E |  |  |  |  | s |
| *Hyalosperma semisterile* | a | N |  |  |  |  | s |
| *Hypochaeris radicata* | a - p | E |  |  |  | nd | s |
| *Maireana lobiflora* | p | N |  |  |  | nd |  |
| *Maireana sp.* | p | N |  |  |  | nd |  |
| *Malva parviflora* | a | E |  |  |  |  | s |
| *Medicago minima* | a - p | E |  |  |  | nd | s |
| *Melaleuca lanceolata* | p | N |  |  |  | nd |  |
| *Moraea setifolia* | p (bulb) | E |  |  | g |  |  |
| *Myoporum platycarpum* | p | N | c |  |  |  |  |
| *Nicotiana goodspeedii* | a - p | N |  |  |  | nd | s |
| *Oxalis perennans* | a | N |  |  |  |  | s |
| *Pimelea micrantha* | p | N |  |  |  | nd |  |
| *Ptilotus seminudus* | p | N |  |  |  | nd |  |
| *Ptilotus spathulatus* | p | N |  |  |  | nd |  |
| *Rhagodia crassifolia-candolleana* intermediate | p | N |  |  |  | nd |  |
| *Rostraria cristata* | a (grass) | E |  |  |  |  | s |
| *Rytidosperma caespitosum* | p (grass) | N |  |  |  | nd | s |
| *Sclerolaena patenticuspis* | p | N |  |  | g |  |  |
| *Senna artemisioides ssp. filifolia* | p | N |  | u |  |  |  |
| *Silene apetala* | a | E |  |  |  |  | s |
| *Sonchus oleraceus* | a | E |  |  |  | nd | s |
| *Teucrium racemosum* | p | N |  |  |  | nd |  |
| *Thysanotus baueri* | p | N |  |  |  | nd |  |
| *Velleia arguta* | p | N |  |  | g |  |  |
| *Vittadinia cuneata* | a - p | N |  |  |  | nd | s |
| *Wurmbea dioica* | p (bulb) | N |  |  |  | nd |  |
| *Zygophyllum apiculatum* | p | N |  | u (patches) |  |  |  |
| *Zygophyllum aurantiacum* | p | N |  | u (patches) |  |  |  |

| **KOOLOOLA** |  |  |  |  |  |  |  |
| --- | --- | --- | --- | --- | --- | --- | --- |
| **Species name** | **annual (a) perennial (p)** | **native (N)**  **exotic (E)** | **canopy dominant (c )** | **understorey dominant (u)** | **groundstorey dominant (g)** | **scattered**  **non-dominant (nd)** | **higher freq season dependent (s)** |
| *Acacia nyssophylla* | p | N |  |  |  | nd |  |
| *Ajuga iva* | p | E |  |  | g |  | s |
| *Amyema preissii* | p | N |  |  |  | nd |  |
| *Atriplex stipitata* | p | N |  | u |  |  |  |
| *Austrostipa sp.* | p (grass) | N |  |  |  | nd |  |
| *Brassica tournefortii* | a - p | E |  |  | g |  |  |
| *Bromus madritensis* | a (grass) | E |  |  |  | nd | s |
| *Calotis hispidula* | a | E |  |  |  | nd | s |
| *Carrichtera annua* | a | E |  |  | g |  |  |
| *Einadia nutans* ssp. *nutans* | p | N |  |  |  | nd |  |
| *Eriochiton sclerolaenoides* | p | N |  |  |  | nd |  |
| *Erodium crinitum* | a - p | E |  |  | g |  | s |
| *Eucalyptus oleosa* | p | N | c (scattered) |  |  |  |  |
| *Euphorbia drummondii* | a | N |  |  |  | nd | s |
| *Geijera linearifolia* | p | N |  |  |  | nd |  |
| *Goodenia pinnatifida* | p | N |  |  |  | nd |  |
| *Heliotropium europaeum* | a | E |  |  |  |  | s |
| *Herniaria cinerea* | a | E |  |  |  |  | s |
| *Hordeum glaucum* | a (grass) | E |  |  |  |  | s |
| *Hypochaeris radicata* | a - p | E |  |  |  | nd | s |
| *Maireana georgei* | p | N |  | u |  |  |  |
| *Maireana sp.* | p | N |  | u |  | nd |  |
| *Marrubium vulgare* | p | E |  | u |  |  |  |
| *Mesembryanthemum crystallinum* | p | E |  |  |  | nd |  |
| *Moraea setifolia* | p (bulb) | E |  |  | g |  |  |
| *Nicotiana glauca* | p | E |  |  |  | nd |  |
| *Rhagodia crassifolia-candolleana* intermediate | p | N |  |  |  | nd |  |
| *Rhagodia parabolica* | p | N |  |  |  | nd |  |
| *Rhagodia spinescens* | p | N |  |  |  | nd |  |
| *Rostraria cristata* | a (grass) | E |  |  |  |  | s |
| *Rytidosperma caespitosa* | p (grass) | N |  |  |  | nd |  |
| *Scabiosa atropurpurea* | a - p | E |  |  |  | nd | s |
| *Schismus barbatus* | a (grass) | E |  |  |  |  | s |
| *Senecio spanomerus* | p | N |  |  |  | nd |  |
| *Sida corrugata* | p | N |  |  | g |  |  |
| *Silene apetala* | a | E |  |  |  |  | s |
| *Sonchus oleraceus* | a | E |  |  |  | nd | s |

| **PORTEE** |  |  |  |  |  |  |  |
| --- | --- | --- | --- | --- | --- | --- | --- |
| **Species name** | **annual (a) perennial (p)** | **native (N)**  **exotic (E)** | **canopy dominant (c )** | **understorey dominant (u)** | **groundstorey dominant (g)** | **scattered**  **non-dominant (nd)** | **higher freq season dependent (s)** |
| *Ajuga iva* | p | E |  |  | g |  |  |
| *Alectryon oleifolius* | p | N | emergent clusters |  | nd |  |  |
| *Asphodelus fistulosus* | p | E | c |  |  |  |  |
| *Austrostipa* sp. | p (grass) | N |  |  |  | nd |  |
| *Brachyscome ciliaris* | a - p | N |  |  |  | nd |  |
| *Brassica tournefortii* | a | E | c |  |  |  |  |
| *Bromus madritensis* | a (grass) | E |  |  |  | nd | s |
| *Calotis hispidula* | a | E |  |  |  | nd | s |
| *Carrichtera annua* | a | E | c |  |  |  |  |
| *Convolvulus angustissimus* ssp*. peninsularum* | p | N |  |  |  | nd |  |
| *Erodium crinitum* | a - p | E |  |  | g |  | s |
| *Eucalyptus oleosa* | p | N | highly scattered |  |  | nd |  |
| *Euphorbia drummondii* | a | N |  |  |  | nd | s |
| *Heliotropium europaeum* | a | E |  |  |  |  | s |
| *Herniaria cinerea* | a | E |  |  |  |  | s |
| *Hordeum glaucum* | a (grass) | E |  |  |  |  | s |
| *Hypochaeris radicata* | a - p | E |  |  |  | nd | s |
| *Moraea setifolia* | p (bulb) | E | c |  |  |  |  |
| *Myoporum platycarpum* | p | N | highly scattered |  |  | nd |  |
| *Rostraria cristata* | a (grass) | E |  |  |  | nd | s |
| *Rytidosperma caespitosa* | p (grass) | N |  |  |  | nd | s |
| *Silene apetala* | a | E |  |  |  | nd | s |
| *Sonchus oleraceus* | a | E |  |  |  | nd | s |
| *Zygophyllum apiculatum* | p | N |  | u (patches) |  | nd |  |
| *Zygophyllum aurantiacum* | p | N |  | u (patches) |  | nd |  |
