# Supplementary material for: Dual-locus DNA metabarcoding reveals southern hairy-nosed wombats (Lasiorhinus latifrons Owen) have a summer diet dominated by toxic invasive plants
Source: PLoS One. 2020 Mar 6;15(3):e0229390. doi: 10.1371/journal.pone.0229390 (PMC7059939; doi:10.1371/journal.pone.0229390)
Supplement: S4 Table — (DOCX) [file pone.0229390.s005.docx]

**S4 Table.** Clinical and pathological findings of post-mortum examinations of Southern Hairy Nosed Wombats (SHNW) recovered from the Murraylands region of South Australia.

| **Reference** | **Origin** | **Age** | **Sex** | **BCS** | **Significant pathological findings** |
| --- | --- | --- | --- | --- | --- |
| **11-0037** | Portee Station | A | F | 1/5 | Skin: Generalised multifocal bilateral alopecia; mild orthokeratotic hyperkeratosis;  Liver: mild fibrous interlobar and diaphragmatic adhesions; Moderate periportal to bridging fibrosis and bile ductular hyperplasia, mild hepatocytic anisocytosis, anisokaryosis and pigmentation.  Brain, brain stem and pons: Moderate to marked neuronal and astrocytic cytoplasmic pigmentation |
| **11-0046** | Portee Station | SA | F | 1.5/5 | Skin: Orange coat discoloration and extensive alopecia of dorsal and body surfaces and lateral hind and fore limbs; photosensitive dermatitis.  Liver Cholestatic hepatitis/ hepatopathy (Multifocal necrosuppurative hepatitis, megalocytosis with marked bile duct proliferation, nodular regeneration and multifocal to diffuse fibrosis) |
| **11-0047** | Portee Station | A | F | 2.5/5 | Skin: orange coat discoloration; moderate dorsal and lateral alopecia with seborrhoea and exudative dermatitis;: mild acanthosis with orthokeratotic hyperkeratosis, mild non suppurative interstitial dermatitis.  Liver: mild to moderate periportal fibrosis and bile duct hyperplasia; mild hepatocellular anisocytosis and anisokaryosis |
| **11-0048** | Portee Station | A | F | 2/5 | Skin: moderate dorsal and lateral alopecia with mild seborrhoea and exudative dermatitis; mild acanthosis with orthokeratotic hyperkeratosis, mild non suppurative interstitial dermatitis  Liver: gall bladder wall thickening; mild to moderate periportal fibrosis and bile duct hyperplasia; mild hepatocellular anisocytosis and anisokaryosis |
| **11-0049** | Portee Station | SA | F | 1/5 | Skin: Orange coat discoloration, severe bilaterally symmetrical extensive alopecia, photosensitive dermatitis.  Liver: moderate megalocytosis, anisocytosis and anisokaryosis, sinusoidal disruption with multifocal single cell necrosis and necrosuppurative hepatitis |
| **12-0142** | Moorunde | SA | F | 2/5 | No significant findings. |
| **12-0143** | Moorunde | A | F | 2/5 | Skin: Bilaterally symmetrical patchy to diffuse alopecia; mild sarcoptic mange. Liver: Mild to moderate biliary hyperplasia and periportal and subcapsular fibrosis. Minimal to mild hepatocellular anisocytosis and anisokaryosis |
| **12-0144** | Moorunde | A | F | 1.5/5 | Skin: exudative dermatitis over dorsal skull; Flanks and abdomen skin: Mild sarcoptic mange; Periocular region: demodectic mange. |
| **12-0190** | Portee Station | A | F | 1/5 | Emaciated. Bilateral symmetrical patchy to diffuse alopecia over the lateral thorax, flank, and thighs.  Anaemia. Multiple organ atrophy. |
| **12-0366** | Moorunde | A | M | 1/5 | Jaundice. Mild sarcoptic mange. Hepatic atrophy and fibrous diaphragmatic adhesions. |
| **12-0399** | Portee Station | SA | F | 2/5 | Jaundice, subcutaneous oedema and ascites.  Skin: Patchy to diffuse alopecia of rump, forelimbs, lateral and ventral body  Liver: Multifocal necrosuppurative necrosis, bridging fibrosis and nodular regeneration (cirrhosis) and cholestasis; Neutrophilic and proliferative cholecystitis;  Kidney: acute tubular necrosis, suppurative nephritis with intratubular oxalate crystals |
| **13-0646** | Kooloola | A | F | 4/5 | MVA (traumatic injuries). Otherwise healthy. |
| **12-0188** | Kooloola | A | M | 3/5 | MVA (traumatic injuries). Otherwise healthy. |
| **11-0169** | Brookfield | J | F | 2/5 | Skin: Patchy alopecia and yellow coat discolouration, mild sarcoptic mange, fungal dermatitis. Enteric coccidiosis; Anaemia, hypoproteinaemia; Ascites and pulmonary oedema. |
| **13-0158** | Brookfield | A | F | 3/5 | MVA (traumatic injuries). Otherwise healthy. |
| **13-0282** | Brookfield | J | F | 2/5 | Patchy hair loss and yellow coat discolouration. Enteric coccidiosis, hypoproteinaemia, ascites and pulmonary oedema |
| **12-0209** | Blanchetown | A | M | 4/5 | MVA (traumatic injuries). Otherwise healthy. |
| **12-0420** | Blanchetown | SA | F | 4/5 | MVA (traumatic injuries). Otherwise healthy. |
| **13-0317** | Berri | A | F | 3/5 | MVA (traumatic injuries). Otherwise healthy. |
| **12-0304** | Morgan | J | M | 3/5 | Mandibular osteomyelitis (developed whilst in care). |

MVA = motor vehicle accident. BCS = Body condition Score; M = male; F = female; A = adult; SA = sub adult; J = juvenile
