# Supplementary material for: Dual-locus DNA metabarcoding reveals southern hairy-nosed wombats (Lasiorhinus latifrons Owen) have a summer diet dominated by toxic invasive plants
Source: PLoS One. 2020 Mar 6;15(3):e0229390. doi: 10.1371/journal.pone.0229390 (PMC7059939; doi:10.1371/journal.pone.0229390)
Supplement: S5 Table — (DOCX) [file pone.0229390.s006.docx]

**S5 Table.** Glucosinolate content of *Carrichtera annua* green plants, seedlings and dead material/seed debris collected at each of the three study sites

| **Site and sample** | **Gluconapin** | **Glucoerucin** | **Glucoraphenin** | **Total** |
| --- | --- | --- | --- | --- |
| Moorunde |  |  |  |  |
| Green seedlings | 110 | 36 | 4 | 149 |
| Green seedlings | 99 | 40 | 3 | 142 |
| Green plant | 105 | 31 | 3 | 140 |
| Dead material/seed | 11 | 1 | 2 | 14 |
|  |  |  |  |  |
| Kooloola |  |  |  |  |
| Green seedlings | 97 | 37 | 4 | 139 |
| Green seedlings | 97 | 38 | 4 | 139 |
| Dead material/seed | 2 | 1 | 1 | 4 |
| Dead material/seed | 1 | - | - | 2 |
|  |  |  |  |  |
| Portee |  |  |  |  |
| Green seedlings | 84 | 41 | 3 | 128 |
| Green seedlings | 95 | 32 | 3 | 130 |
| Dead material/seed | 12 | 1 | 4 | 17 |
| Dead material/seed | 16 | 1 | 4 | 21 |
|  |  |  |  |  |

^a^ Each value is a mean of two replicates and standard deviations do not exceed 15%.

^b^All values are given in µmol/g DW.
