# Supplementary material for: Dual-locus DNA metabarcoding reveals southern hairy-nosed wombats (Lasiorhinus latifrons Owen) have a summer diet dominated by toxic invasive plants
Source: PLoS One. 2020 Mar 6;15(3):e0229390. doi: 10.1371/journal.pone.0229390 (PMC7059939; doi:10.1371/journal.pone.0229390)
Supplement: S1 Fig — Unrooted UPGMA trees of reference DNA barcode sequences of plant species collected from three sites in the Murraylands of South Australia (Moorunde, Kooloola, Portee) generated using Geneious 8.0 (www.geneious.com, Kearse et al. 2012. Bioinformatics 28:1647–1649). Panel A represents separation of ndhJ sequences. Panel B represents separation of rbcL sequences. (PDF) [file pone.0229390.s007.pdf]

S1 Fig. UPGMA trees of reference barcodes. Unrooted UPGMA trees of reference DNA barcode sequences of plant species collected from three sites in the Murraylands of South Australia (Moorunde, Kooloola, Portee) generated using Geneious 8.0 (www.geneious.com, Kearse et al. 2012. Bioinformatics **28**:1647-1649). Panel A represents separation of *ndh*J sequences. Panel B represents separation of *rbc*L sequences.

A.

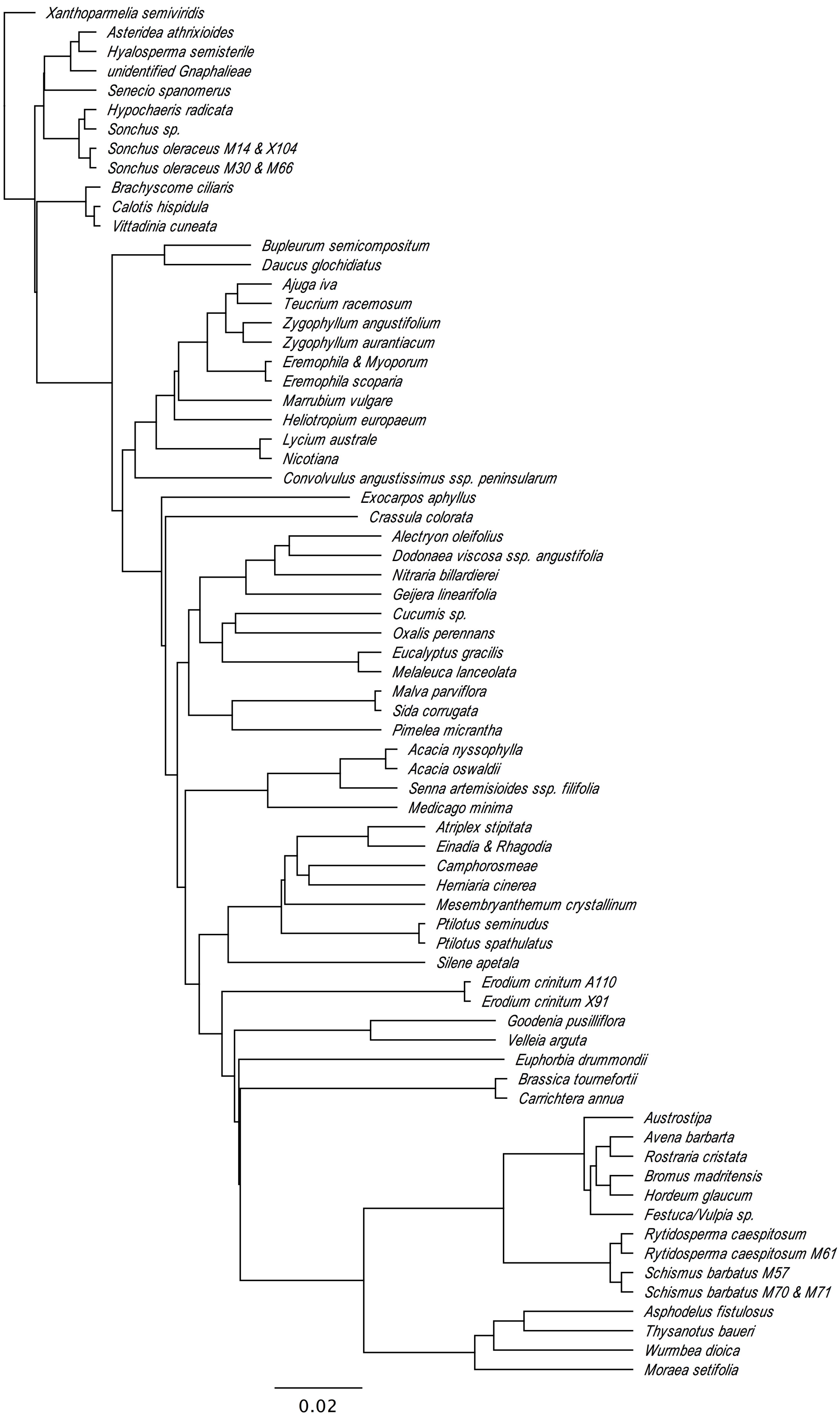

B.

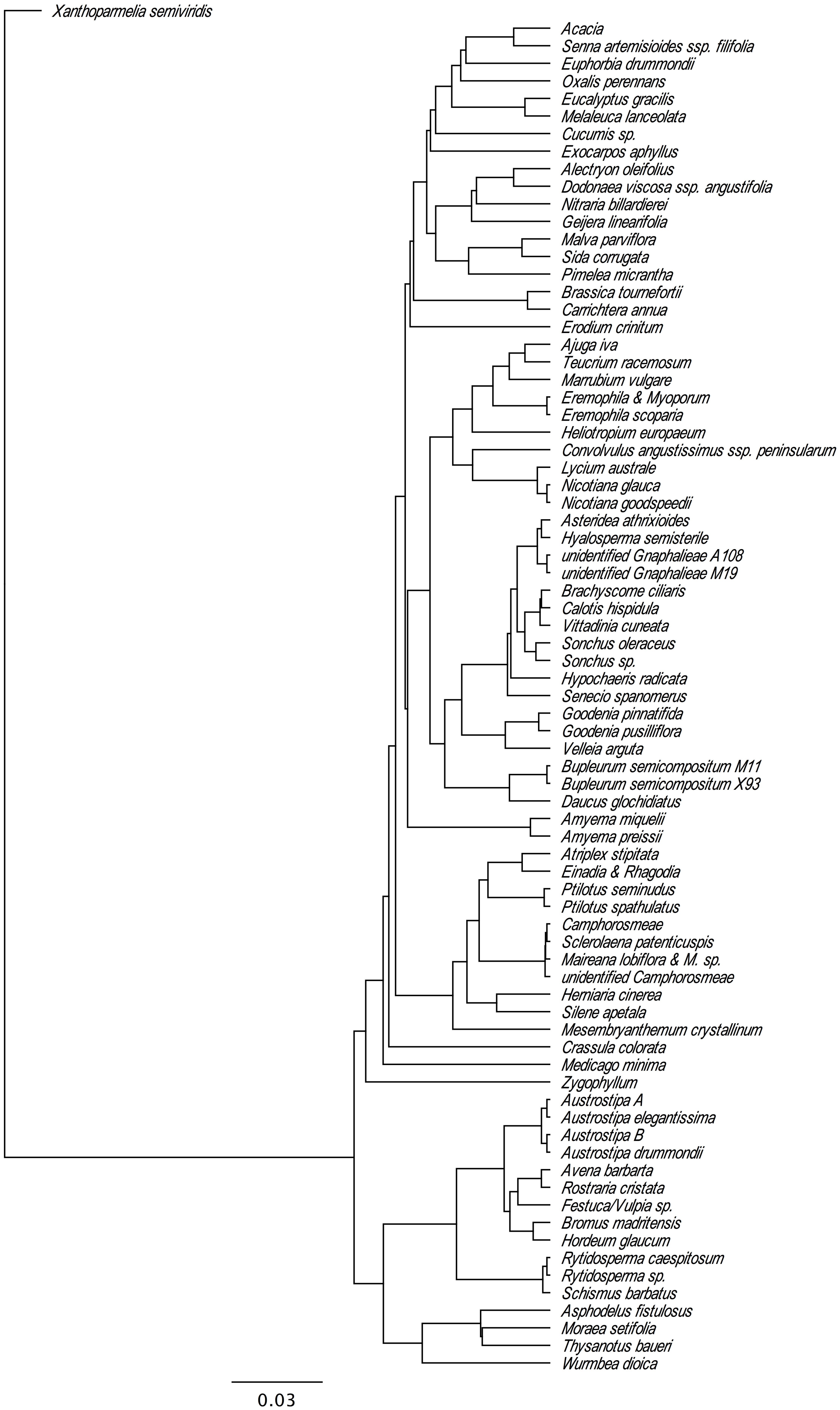

Xanthoparmelia semiviridis
Acacia
Senna artemisioides ssp. filifolia
Euphorbia drummondii
Oxalis perennans
Eucalyptus gracilis
Melaleuca lanceolata
Cucumis sp.
Exocarpos aphyllus
Alectryon oleifolius
Dodonaea viscosa ssp. angustifolia
Nitraria billardierei
Geijera linearifolia
Malva parviflora
Sida corrugata
Pimelea micrantha
Brassica tournefortii
Carrichtera annua
Erodium crinitum
Ajuga iva
Teucrium racemosum
Marrubium vulgare
Eremophila & Myoporum
Eremophila scoparia
Heliotropium europaeum
Convolvulus angustissimus ssp. peninsularum
Lycium australe
Nicotiana glauca
Nicotiana goodspeedii
Asteridea athrixioides
Hyalosperma semisterile
unidentified Gnaphalieae A108
unidentified Gnaphalieae M19
Brachyscome ciliaris
Calotis hispidula
Vittadinia cuneata
Sonchus oleraceus
Sonchus sp.
Hypochaeris radicata
Senecio spanomerus
Goodenia pinnatifida
Goodenia pusilliflora
Velleia arguta
Bupleurum semicompositum M11
Bupleurum semicompositum X93
Daucus glochidiatus
Amyema miquelii
Amyema preissii
Atriplex stipitata
Einadia & Rhagodia
Ptilotus seminudus
Ptilotus spathulatus
Camphorosmeae
Sclerolaena patenticuspis
Maireana lobiflora & M. sp.
unidentified Camphorosmeae
Herniaria cinerea
Silene apetala
Mesembryanthemum crystallinum
Crassula colorata
Medicago minima
Zygophyllum
Austrostipa A
Austrostipa elegantissima
Austrostipa B
Austrostipa drummondii
Avena barbarta
Rostraria cristata
Festuca/Vulpia sp.
Bromus madritensis
Hordeum glaucum
Rytidosperma caespitosum
Rytidosperma sp.
Schismus barbatus
Asphodelus fistulosus
Moraea setifolia
Thysanotus baueri
Wurmbea dioica
0.03
